# Clinical, Immunological, and Genetic Features in 49 Patients With ZAP-70 Deficiency: A Systematic Review

**DOI:** 10.3389/fimmu.2020.00831

**Published:** 2020-05-05

**Authors:** Niusha Sharifinejad, Mahnaz Jamee, Majid Zaki-Dizaji, Bernice Lo, Mohammadreza Shaghaghi, Hamed Mohammadi, Farhad Jadidi-Niaragh, Shiva Shaghaghi, Reza Yazdani, Hassan Abolhassani, Asghar Aghamohammadi, Gholamreza Azizi

**Affiliations:** ^1^Student Research Committee, Alborz University of Medical Sciences, Karaj, Iran; ^2^Alborz Office of USERN, Universal Scientific Education and Research Network (USERN), Alborz University of Medical Sciences, Karaj, Iran; ^3^Legal Medicine Research Center, Legal Medicine Organization, Tehran, Iran; ^4^Sidra Medicine, Division of Translational Medicine, Research Branch, Doha, Qatar; ^5^Johns Hopkins Hospital, Baltimore, MD, United States; ^6^Research Center for Immunodeficiencies, Children's Medical Center, Tehran University of Medical Sciences, Tehran, Iran; ^7^Non-Communicable Diseases Research Center, Alborz University of Medical Sciences, Karaj, Iran; ^8^Department of Immunology, School of Medicine, Alborz University of Medical Sciences, Karaj, Iran; ^9^Immunology Research Center, Tabriz University of Medical Sciences, Tabriz, Iran; ^10^Department of Immunology, School of Medicine, Tabriz University of Medical Sciences, Tabriz, Iran; ^11^Division of Clinical Immunology, Department of Laboratory Medicine, Karolinska Institute at Karolinska University Hospital Huddinge, Stockholm, Sweden

**Keywords:** Primary Immunodeficiency, combined immunodeficiency, *ZAP70* mutation, ZAP-70 deficiency, CD8^+^ T cell lymphopenia

## Abstract

**Background:** Zeta-Chain Associated Protein Kinase 70 kDa (ZAP-70) deficiency is a rare combined immunodeficiency (CID) caused by recessive homozygous/compound heterozygous loss-of-function mutations in the *ZAP70* gene. Patients with ZAP-70 deficiency present with a variety of clinical manifestations, particularly recurrent respiratory infections and cutaneous involvements. Therefore, a systematic review of ZAP-70 deficiency is helpful to achieve a comprehensive view of this disease.

**Methods:** We searched PubMed, Web of Science, and Scopus databases for all reported ZAP-70 deficient patients and screened against the described eligibility criteria. A total of 49 ZAP-70 deficient patients were identified from 33 articles. For all patients, demographic, clinical, immunologic, and molecular data were collected.

**Results:** ZAP-70 deficient patients have been reported in the literature with a broad spectrum of clinical manifestations including recurrent respiratory infections (81.8%), cutaneous involvement (57.9%), lymphoproliferation (32.4%), autoimmunity (19.4%), enteropathy (18.4%), and increased risk of malignancies (8.1%). The predominant immunologic phenotype was low CD8+ T cell counts (97.9%). Immunologic profiling showed defective antibody production (57%) and decreased lymphocyte responses to mitogenic stimuli such as phytohemagglutinin (PHA) (95%). Mutations of the *ZAP70* gene were located throughout the gene, and there was no mutational hotspot. However, most of the mutations were located in the kinase domain. Hematopoietic stem cell transplantation (HSCT) was applied as the major curative treatment in 25 (51%) of the patients, 18 patients survived transplantation, while two patients died and three required a second transplant in order to achieve full remission.

**Conclusion:** Newborns with consanguineous parents, positive family history of CID, and low CD8+ T cell counts should be considered for ZAP-70 deficiency screening, since early diagnosis and treatment with HSCT can lead to a more favorable outcome. Based on the current evidence, there is no genotype-phenotype correlation in ZAP-70 deficient patients.

## Introduction

**Figure 1:**
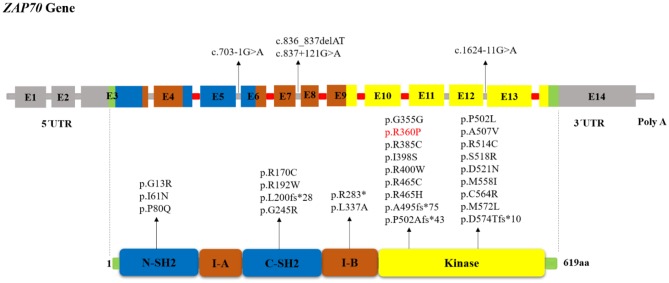
Schematic structure of the ZAP70 gene and location of reported mutations in patients with ZAP-70 deficiency. The indicated ZAP-70 domains are the amino-terminal SH2 domain (N-SH2), interdomain A (I-A), carboxy-terminal SH2 domain (C-SH2), interdomain B (I-B), and the kinase domain. The disease-causative mutations in ZAP-70 deficiency occur throughout the full-length gene without obvious hotspots although the majority of mutations resided within the Kinase domain. Introns that interrupted codons are marked in red. In the cases in which the mutation's effect on the protein (except for splice site and long InDel mutations) had not been reported, we used MutationTaster software (http://www.mutationtaster.org) to predict amino acid changes. The red colored mutation indicates gain of function mutation.

Protein-tyrosine kinases (PTKs) are known to have an integral role in T cell activation. Activation of T-cell antigen receptor (TCR) leads to tyrosine phosphorylation of a number of cellular proteins including Zeta(ζ)-Chain Associated Protein Kinase 70 kDa (ZAP-70), a member of the Syk family (non-receptor protein tyrosine kinase family), that co-precipitates with zeta upon TCR stimulation (1). The *ZAP70* gene consists of the kinase domain, Src homology 2 (SH2)-kinase linker, inter-SH2 linker, and two SH2 domains (Figure 1). Activated ZAP70 regulates motility, adhesion and cytokine expression of specific lymphocytes, mainly γδT-cells, memory CD8^+^ T-cells, NK-cells, MAIT T-cells, naive CD8^+^ T-cell, regulatory T-cells, memory CD4^+^ T-cells, and naive CD4^+^ T-cells. Indirectly, this protein contributes also to the development and activation of B cells. Biallelic mutations in the *ZAP70* gene result in unstable or abnormal protein expression. Deficiency of ZAP-70 causes a combined immunodeficiency (CID), presenting with recurrent infections, slightly milder than those with recessive forms of severe CID (SCID) (2). Patients with SCID usually develop failure to thrive, persistent diarrhea, respiratory symptoms, and/or thrush in the first 2–7 months of life. Pneumocystis pneumonia, significant bacterial infections and disseminated *Bacillus Calmette-Guerin (BCG)* infection are common presenting illnesses. Occasionally, there are SCID patients who do not present with failure-to-thrive and thus, are not recognized to have immunodeficiency until late in the first year of life. SCID is fatal in the first 2 years of life unless the patient is treated with extremely restrictive isolation, hematopoietic stem cell transplant or therapy that replaces the abnormal gene or gene product.

Mutations in *ZAP70* were identified initially in patients of Mennonite descent and subsequently in other ethnicities, including Hispanics, Japanese, Kurdish, Turkish, Portuguese, Caucasian, Mexican, Malagasy, and Iranian patients (3–9). World map of ZAP-70 deficient patients is available in Figure S1. ZAP-70 deficiency presents with a history of recurrent opportunistic infections, although, *Pneumocystis jirovecii* pneumonia and *Cytomegalovirus (CMV)* pneumonitis are less common (10). Autoimmunity or manifestations of immune dysregulation such as ulcerative colitis and blood cytopenias (11), pustular skin lesions and subcutaneous nodules (12), lymphoma (13), Omenn syndrome, and hemophagocytic lymphohistiocytosis (HLH) (14) have also been reported. Patients with ZAP-70 deficiency often present with normal to elevated numbers of circulating lymphocytes, including B cells, CD3^+^, and CD4^+^ T cells, but an absence of CD8^+^ T cells in the peripheral blood. All patients have normal or reduced serum immunoglobulin (Ig) G, while IgM and IgA levels are often normal (15). Specific antibody production is variable, with some patients having normal tetanus antibodies or specific immunoglobulin E (IgE) antibodies against allergens (2, 16, 17).

Here, we systematically reviewed the clinical, immunological and genetic features of patients with ZAP-70 deficiency in order to achieve a comprehensive view of this disease.

## Methods

### Search Strategy

A comprehensive search, limited to articles written in the English language, was performed using PubMed, Web of Science, and Scopus databases, applying the following search terms: “Zeta chain-associated protein of 70 kilo Daltons” or “Zeta chain-associated Protein Tyrosine Kinase” or “Zap-70” or “ZAP70” or “ZAP70 mutation” or “ZAP-70 Protein Tyrosine Kinase” or “ZAP-70 deficiency”; combined with the following search terms, using “AND” command: “Immunodeficiency” or “Severe Combined Immunodeficiency” or “SCID” or “Combined Immunodeficiency” or “CID” or “Primary Immunodeficiency” or “PID” or “lymphopenia” or “CD8+ T-cell lymphopenia” or “hypomorphic mutations in severe combined immunodeficiency disease.” The search was conducted using these terms in the keywords, titles, and abstracts. Reference lists of all full-text articles and major reviews identified in this search were hand-searched for additional studies.

### Study Selection

The articles were first screened based on the title and abstract to exclude all irrelevant studies and were categorized into three groups (include, exclude, or unclear); the full-text version of all “unclear” articles was checked and subsequently classified in one of the two categories (include or exclude). All full-text manuscripts were assessed for eligibility criteria: written in English, conducted on human subjects, reporting at least one patient with ZAP-70 deficiency diagnosis, and detailed description of epidemiological, clinical, and immunological features associated with genetic mutations. Studies using animal models, reviews, congress abstracts, and articles in languages other than English were excluded. When necessary, the corresponding authors were contacted.

### Data Extraction

In the first step, two researchers extracted the data from the studies included. The following data were collected from each article: publication year, the number of participants, and demographic, clinical, laboratory, and molecular data of the patients. The evaluation of immunologic data quality was based on the age-matched normal ranges and the normal ranges included in each article. Those patients appearing in more than one publication were identified and the duplicate data was removed. Two reviewers performed the selection process independently, while the third reviewer was consulted to resolve disagreements between two reviewers.

### Statistical Analysis

Central and descriptive statistics were reported for quantitative data. For variables with abnormal distribution, median and interquartile ranges (IQR) were reported. Kaplan–Meier curve and log-rank test were utilized to compare different survival estimates. All statistical analyses were performed using the SPSS software (v. 25.0, Chicago, IL).

## Results

### Study Characteristics

**Figure 2:**
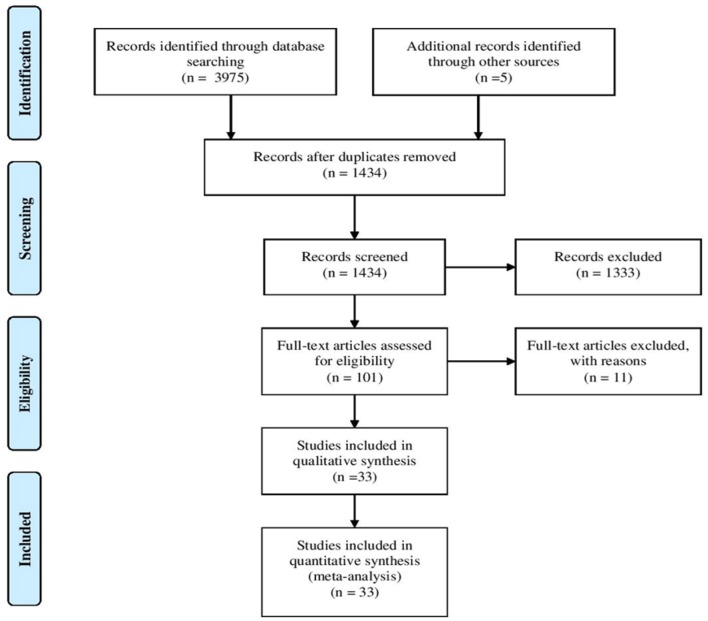
Flowchart of the systematic search and study selection process.

The literature search identified a total of 3,975 articles. Two thousand, five hundred forty-six articles were duplicated and 1,333 articles were excluded following title and abstract screening. Furthermore, eleven articles were excluded as they only reported general information regarding ZAP-70 deficient patients lacking specific details. As shown in Figure 2, 33 articles fulfilled the inclusion criteria and were subsequently used for data extraction. A total of 131 ZAP-70 deficient patients were reported in these 33 articles and after removing overlapping cases, 49 unique patients remained for data analysis (3–9, 12–14, 17–39).

### Epidemiologic Characteristics of ZAP-70 Deficient Patients

In this systematic review, we included 49 ZAP-70 deficient patients (27 males, 20 females, and 2 with unknown gender) (21). Most of the patients were of Mennonite (30.6%) descent, followed by Turkish (22.4%), Japanese and Caucasian ethnicity (each 6.1%). The median (IQR) age of diagnosis was 10.4 (7.0–18.7) months with a median (IQR) diagnostic delay of 5 (1.2–11.5) months. A positive family history of immunodeficiency was reported in 25 cases (61%). The demographic data of all patients are summarized in Table 1. Thirty-five patients (76.1%) were alive at the time of the report, 11 (23.9%) were deceased, and the life status for 3 patients was not mentioned (21, 25). Acute respiratory distress syndrome, CMV pneumonitis, multiorgan failure due to secondary hemophagocytic syndrome, disseminated intravascular coagulation, recurrent breathing arrest, and atrioventricular block were the reported causes of death.

**Table 1:** Demographic data of patients with ZAP-70 deficiency.

**Parameters (no. of evaluated patients)**	**Results**
Sex ratio, Male/Female, (*n* = 47)	27/20
Consanguinity, (*n* = 36) (%), 1st degree-2nd degree	13 (36.1)−6 (16.7)
Familial case, (*n* = 41) (%)	25 (61)
Dead, (*n* = 42) (%)	11 (23.9)
Age at onset (m), median (IQR), (*n* = 38)	4.0 (2.0–7.0)
Age of genetic diagnosis (m), median (IQR), (*n* = 12)^*^	7.5 (4.5–22.2)
Delay in diagnosis (m), median (IQR), (*n* = 28)	5.0 (1.2–11.5)
Time (m) of follow-up since birth, median (IQR), (*n* = 36)	39.0 (17.25–97.5)
Age at presentation of infection (m), median (IQR), (*n* = 20)	4.5 (2.2–6.7)
Age of first cutaneous manifestation (m), median (IQR), (*n* = 12)	3.5 (2.0–11.3)
Age at presentation of autoimmunity (m), median (IQR), (*n* = 7)	8.0 (2.0–9.0)
Age at presentation of diarrhea (m), median (IQR), (*n* = 7)	5.0 (3–7)
Age at presentation of lymphoproliferative (m), median (IQR), (*n* = 5)	11 (5–16.0)
Age of death (m), median (IQR), (*n* = 9)	18.0 (13.5–24.5)

*The median is shown (IQR, with 25th and 75th percentiles). N, count; m, month*.

* *Age of genetic diagnosis was mentioned without reporting the age of onset in some cases*.

### Molecular Findings

The human *ZAP70* gene is located on chromosome position 2q11.2 (17). It contains 2 non-coding and 12 coding exons that encode a 619-amino acid protein, ZAP-70. Structurally ZAP70 is composed of two SH2 domains and a carboxy-terminal kinase domain that are separated by inter-domain A and B (40). As shown in Figure 1, the rare *ZAP70* loss-of-function mutations, which abolish ZAP-70 expression and result in SCID phenotype, are mostly located in the kinase domain (41). Among the 49 patients (41 families) identified in our study, 38 (77.5%) patients (in 33 families) had homozygous mutations, 10 (16.3%) patients (in 8 families) had compound heterozygous *ZAP70* mutations, and for one patient, the site and type of mutation was not reported (26). Overall, 32 different mutations were detected. These variants consisted of 23 missense, 5 indel-frameshift, 3 splice site, and 1 nonsense. Mutations of the *ZAP70* gene were located throughout the gene, and there did not appear to be any mutational hotspot (Figure 1). The splice site mutation c.1624-11G>A (p.K541_K542insLEQ) was the most frequent mutation, as it was identified in 10 different families most of which were of Mennonite descent. Of the 12 families of Mennonite descent, 9 of them had homozygous c.1624-11G>A mutations and 2 families had compound heterozygous c.1763C>A/ c.1624-11G>A mutations.

Most of the reported disease mutations in the *ZAP70* gene were homozygous recessive or compound heterozygous loss-of-function mutations with abolished protein expression and/or disrupted catalytic activity but with normal mRNA expression (7). However, a sibling pair, who only presented with early-onset autoimmune diseases and no opportunistic infections, was reported recently to be compound heterozygous for a loss and a gain-of-function mutation (5). In these patients, the combination of the *ZAP70* R192W allele with decreased ζ-chain binding plus the constitutively active R360P allele with decreased auto-inhibition led to the development of autoimmunity. To analyze genotype-phenotype correlation, the patients were divided into three categories: 1) “classical” group of 36 patients with amorphic mutations that abolish protein expression and/or function, 2) “leaky” group consisting of 5 patients with hypomorphic mutations that result in residual ZAP70 protein expression (8, 27, 36–38), 3) “atypical” group including 2 patients with both gain and loss-of-function mutations (5). For other patients we did not have enough information about protein expression or function of ZAP70 to categorize them. We did not find any significant correlation between the clinical and laboratory findings in the classical and leaky groups nor among the different mutation types. Table S1 represents the reported *ZAP70* gene mutations in patients with ZAP-70 deficiency.

### Clinical Findings

**Figure 3:**
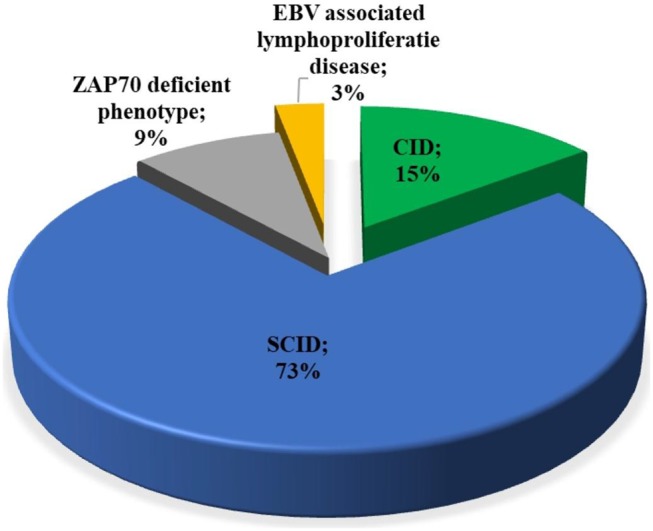
Distribution of the available primary clinical diagnosis of the patients with ZAP-70 deficiency. EBV, Epstein-Barr virus; SCID, severe combined immunodeficiency; CID, combined immunodeficiency.

**Figure 4:**
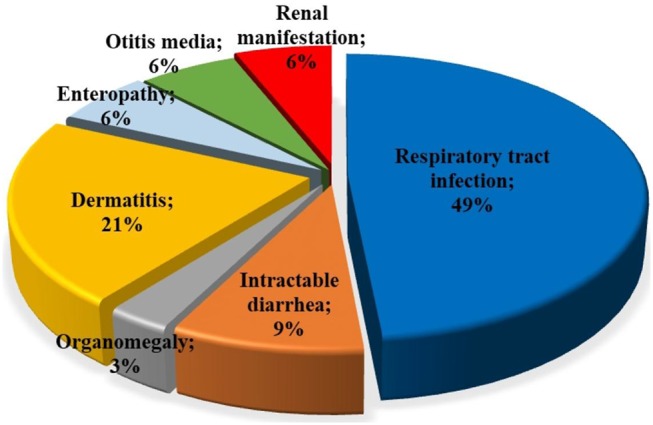
Type of first clinical manifestations in patients with ZAP-70 deficiency. The most common first presentations in ZAP-70 deficient patients reported were respiratory tract infections and dermatitis.

Among patients with initial clinical diagnosis (*n* = 34) reported, most patients had a SCID clinical diagnosis (*n* = 25, 73.5%). Figure 3 shows the initial clinical diagnosis of ZAP-70 deficient patients before genetic evaluation. As presented in Figure 4, respiratory tract infection was the most common first presenting feature (*n* = 16, 48.5%) followed by dermatitis (*n* = 7, 21.2%). Cutaneous manifestations were found to be the earliest and lymphoproliferative disorder the latest clinical presentation, with median (IQR) age of 3.5 (2.0–11.3) and 11 (5–16.0) months, respectively.

#### Infectious Complications

Pneumonia (*n* = 30, 71.4%), diarrhea (*n* = 19, 50%), and upper respiratory tract infections [sinusitis (*n* = 3, 8.3%) and other ENT (Ear, Nose, Throat) infections (*n* = 7, 19.4%)], including otitis media, bilateral otitis, and acute nasopharyngitis, were the most common infectious manifestations (Table 2). The most common infection-causing agents identified in all patients were viruses (*n* = 24, 63.2%) (particularly *CMV* [29%], *Varicella* [29%], *Epstein-Barr virus* [12.5%], and *Rotavirus* [4.1%]), bacteria (*n* = 14, 36.8%) (mainly *BCG* [18.4%]), fungal pathogens (*n* = 14, 38.9%) [predominantly *Candida albicans* (28.9%)], and protozoa (*n* = 13, 33.3%) (especially *Pneumocystis carinii* [24.5%]). One patient developed lung tuberculosis at the age of 48 months (33), and another patient had complications of disseminated mycobacterial disease at the age of 13 months without previous complication with BCG vaccination (34). Two cousins were also reported to have left axillary lymphadenitis due to BCG vaccinations at the maternity hospital (4). Infectious cutaneous presentations were reported in 3 patients (P17- P26- P47) and included disseminated *Molluscum contagiosum*, oral and cutaneous warts, and varicella zoster virus (VZV) dermatitis (27, 32, 36). A patient was described to have fungal abscess (P29) (33), another patient developed viral cerebellitis (P31) (34), and one patient succumbed to VZV encephalitis (P27) (32).

**Table 2:** Clinical manifestations of patients with ZAP-70 deficiency.

**Parameters**	**No. patients**	**Frequency**	**Patients coding**	**References**
Pneumonia (%)	42	30 (71.4)	P1- P2- P4- P5- P10- P11- P14 to 20- P22 to 27- P30- P33 to35- P42 to 44- P46 to 49	(3, 4, 6, 7, 9, 13, 14, 17, 20, 23, 24, 27–33, 35–39)
Diarrhea (%)	38	19 (50)	P2- P4- P5- P6- P13- P14- P20- P26- P28- P30-P33- P37- P38- P41- P43 to 46- P49	(3, 5–7, 9, 13, 14, 17, 22, 26, 32, 33, 35, 38, 39)
Sinusitis (%)	36	3 (8.3)	P17- P28- P43	(27, 33, 39)
Ear, nose, throat problem (%)	36	7 (19.4)	P1- P2- P17- P19- P34- P43- P47	(4, 6, 17, 20, 27, 36, 39)
Bronchiectasis (%)	37	3 (8.1)	P17- P23- P47	(27, 30, 36)
Viral cerebellitis	36	1 (2.7)	P31	(34)
VZV encephalitis	36	1 (2.7)	P27	(32, 33)
Septicemia (%)	36	4 (11.1)	P26- P27- P43	(7, 32, 39)
Abscess	36	1 (2.8)	P29	(33)
Candidiasis (%)	38	11 (28.9)	P2- P5- P14- P16- P17- P27- P30- P34- P38-P42- P43	(3, 6, 14, 17, 27, 32, 33, 37, 39)
Bacillus Calmette-Guerin (BCG)osis (%)	38	7 (18.4)	P18-P19-P30-P31-P44-P49	(4, 9, 33–35)
Skin manifestation (%)	38	22 (57.9)	P2-P7-P14-P16 to 19-P24-P26-P27-P34-P35-P37-P40 to 47-P49	(4–7, 9, 12, 14, 17, 27, 29, 32, 35–39)
Hematologic abnormality (%)	36	6 (16.7)	P14-P24-P29-P39-P40-P43	(5, 6, 14, 29, 33, 39)
Neurologic abnormality (%)	36	5 (13.9)	P27-P29-P30-P31-P48	(8, 33, 34)
Renal abnormality (%)	36	4 (11.1)	P29-P40 to 42	(5, 33, 37)
Cardiovascular abnormality (%)	36	2 (5.6)	P29-P48	(8, 33)
Hepatobiliary (%)	36	1 (2.8)	P22	(28)
Autoimmunity (%)	36	7 (19.4)	P29-P39 to 43-P46	(5, 6, 33, 37–39)
Failure to thrive (%)	39	17 (43.6)	P4- P5- P6- P13- P14- P18- P19- P23- P27- P30-P35- P37- P38- P41 to 43- P49	(3–6, 9, 14, 17, 22, 26, 30, 32, 33, 37, 39)
Entropathy (%)	38	7 (18.4)	P6- P40 to 42- P44- P46- P49	(5, 9, 22, 35, 37, 38)
Hepatomegaly (%)	36	7 (19.4)	P14- P16- P20- P24- P28- P30- P31	(13, 14, 29, 33, 34)
Splenomegaly (%)	36	5 (13.9)	P16- P20- P24- P28- P48	(13, 14, 29, 33)
Lymphadenopathy (%)	37	12 (32.4)	P4- P14 to 26- P18 to 20- P31- P42- P46- P48-P49	(4, 8, 9, 13, 14, 17, 34, 37, 38)
Malignancy (%)	37	3 (8.1)	P11- P20- P29	(13, 24, 33)

#### Non-infectious Complications

Different types of non-infectious cutaneous manifestations, including erythematous urticarial rash, bullous pemphigoid, eczema perineal ulcers, widespread xerosis, ichthyosis, and subcutaneous nodule, were described in 19 (38.8%) patients. Hematologic disorders, consisting of non-immune hemolytic anemia, antibody to clotting factor VIII, hemophagocytic syndrome, immune thrombocytopenic purpura (ITP), autoimmune hemolytic anemia (AIHA), and bicytopenia, were reported in 6 (16.7%) patients. Neurologic involvements (*n* = 5, 13.9%) included types of silent brain infarct (2 patients), VZV encephalitis (1 patient), viral cerebellitis (1 patient), and facial paralysis (1 patient). Renal involvement (11.1%) in ZAP70 deficiency consisted of nephrotic syndrome (2 patients), one case of proteinuria with lack of progression to nephrotic syndrome, and IgA nephropathy (1 patient). Two patients (5.6%) were found to have cardiovascular problems, which manifested as persistent hypertension and atrioventricular block (8, 33). One patient developed cholestatic liver disease (P22) (28).

Autoimmunity (*n* = 7, 19.4%) was mentioned in different categories as autoimmune cytopenia (*n* = 2), autoimmune nephritis (*n* = 2), autoimmune enteropathy (*n* = 1), dermal involvements in the form of bullous pemphigoid (*n* = 1), and adrenal insufficiency (*n* = 1). Polyautoimmunity was present in 2 siblings, one with bullous pemphigoid, nephrotic syndrome, antibody to clotting factor VIII, and inflammatory bowel disease (P40) and a second patient with bullous pemphigoid and inflammatory bowel disease (P41). Both patients with polyautoimmunity had compound heterozygous mutations: R192W (LOF) and R360P (GOF), producing a weakly hyperactive ZAP70 protein (5).

Enteropathy (18.4%) and failure to thrive (43.6%) were other reported manifestations. Among patients with lymphoproliferative diseases, lymphadenopathy was the most frequent manifestation (32.4%), followed by hepatomegaly (19.4%), and splenomegaly (13.9%). Enlarged tonsils were also reported in 3 cases. **Three** patients (8.1%) developed malignant diseases (Table 2) including EBV-associated diffuse large B-cell lymphoma, non-EBV-associated large B cell lymphoma, and non-Hodgkin lymphoma.

### Immunological Findings

Immunological data are summarized in Table 3. Of the evaluated patients, 11.1% had lymphopenia; low CD8^+^ cell counts were the most common profile (97.9%), followed by low numbers of NK cells (23.8%) and CD19^+^ B cells (11.1%). CD4^+^ T cell deficiency was reported in 12.2% of the ZAP-70 deficient patients. However, one patient was reported to have elevated CD8+ level, which deviates from the usual immunologic findings for ZAP-70 deficiency (9). Hypogammaglobulinemia and hyper IgM phenotype were each noted in 3 (13%) patients (3, 8, 22, 34, 38, 42). Adjusted to the age-matched normal ranges or the normal ranges included in each article, serum immunoglobulin levels were mostly reported to be normal, but some cases showed decreased levels of IgG (*n* = 12 out of 44, 27.3%), IgA (*n* = 6 out of 45, 13.3%), and IgM (*n* = 5 out of 45, 11.1%). A decline in antibody response to polysaccharide and peptide vaccine was detected in 5 (55.6%) and 16 (80%) patients, respectively. Reduction in T-cell receptor excision circles (TREC) levels and phytohemagglutinin (PHA) response were observed in 5 (50%) and 38 (95%) of the cases with available data, respectively (5, 7, 8, 27, 36–38). A supplementary file including each individual clinical, immunological, and genetic features is available as Supplementary File (Table 1_V1).

**Table 3:** Immunologic features of ZAP-70 deficiency at the time of diagnosis.

**Parameters**	**No. patients with available data**	**Results**
WBC (×10^3^ Cell/μL), median (IQR)	9	13.6 (10.9–26.9)
ALC ×10^3^ (cells/μL), median (IQR)	30	5.4 (3.5–8.2)
CD3+ T cells (cell/μL), median (IQR)	32	2,516 (1,565–3,978)
CD4+ T cells (cell/μL), median (IQR)	32	2,312 (1,595–3,320)
CD8+ T cells (cell/μL), median (IQR)	35	75 (42–233)
NK cell, (cell/μL), median (IQR)	16	417 (134–897)
CD19+ B cells (cell/μL), median (IQR)	29	1,241 (511–1,869)
IgG, mg/dL, median (IQR)	37	570 (230–955)
IgA (mg/dL), median (IQR)	37	100 (38–154)
IgM (mg/dL), median (IQR)	37	109 (64–159)
IgE (U/ml), median (IQR)	15	51 (6–218)
PHA response, median (IQR)	20	56 (1.7–602)
Decreased PHA response (%)	40	38 (95%)
TREC, median (IQR)	8	69.5 (14.5–95.75)
Decreased TREC (%)	10	5 (50%)

*The median is shown (IQR, with 25th and 75th percentiles)*.

*ALC, absolute lymphocytes counts; Ig, immunoglobulin; WBC, white blood cell; NK cell, natural killer cell; PHA, phytohemagglutinin; TREC, T-cell receptor excision circles*.

### Treatment

Twenty-five patients (51%) underwent hematopoietic stem cell transplantation (HSCT), using bone marrow (*n* = 17; 68%), peripheral blood (*n* = 5; 20%), or cord blood (*n* = 3; 12%) as their stem cell source. One of the patients received bone marrow for his first transplant then peripheral blood stem cells during his second transplant (24). Preferred reported conditioning regimens were either toxcicitxy reduced or myeoloablative (7 patients), utilized for HSCT. The three regimens mentioned in the cited literature were: (1) busulfan and cyclophosphamide, (2) busulfan, fludarabine, and anti-thymocyte globulin (ATG), and (3) melphalan, fludarabine, and ATG.

The median age of patients receiving a transplant was 10 (6.7–17.2) months. Among the 21 cases with donor type mentioned, 13 (61.9%) had a matched sibling donor and 8 (38.1%) had a matched unrelated donor. Graft versus host disease (GVHD) (*n* = 9, 36%) and infections (*n* = 4, 16%) were the most commonly reported complications after HSCT. One liver failure (28) and one encephalic lesion in the brainstem and thalamus (32) have also been reported. GVHD prophylaxis was applied in 4 cases, using immunosuppressive drugs such as cyclosporine, methotrexate, and tacrolimus. The follow-up time was reported for 19 patients with a median of 36 (22.8–154.8) months after HSCT. During follow up, engraftment was achieved in 18 patients with their first HSCT and in 3 patients with their second HSCT (P23- P33- P37). Eighteen patients were alive and well after their first transplant, while other patients had a second transplant (*n* = 3) or died (*n* = 2). One patient went through an unsuccessful transplantation (P12) (25) while another patient's transplantation outcome was not mentioned (P32) (6). The Kaplan-Meier survival curve, comparing the prognosis of ZAP-70 deficient patients who underwent HSCT with those who did not, is available in Figure S2. As presented in the plot, the mortality rate was significantly lower in patients who underwent HSCT (*p* < 0.001).

The two deceased patients had received their HSCT at the age of 20 and 30 months old. Three patients at the age of 7, 9, and 18 months needed a second transplant to reach complete engraftment. One patient with an unsuccessful transplantation was 9 months at the age of HSCT. Eight out of twelve patients (66%) with the above mentioned complications post-HSCT were older than 6 months at the time of transplantation.

Steroids and intravenous immunoglobulin (IVIG) have been tried as a prophylactic or to control adverse side effects of transplantation in 8 (32%) and 18 (36.7%) patients, respectively. Among the patients receiving steroids, five were clinically responsive (35).

## Discussion

The present study was designed to systematically review the clinical, immunological, and molecular characteristics of ZAP-70 deficient patients. Parental consanguinity and family history are shown to be a relevant indicator in the suspected patients. According to the statistics, ZAP-70 deficiency is more prevalent in Mennonite ethnicity (30.6%). In fact, it was first described in 3 Mennonite patients (16) and since then has been reported in various cases (6, 22, 31). It has been found that the rates of consanguineous marriages and inbreeding in the Mennonite population have increased during recent centuries (43). Our review illustrated that 97.3% of the patients presented within 12 months of age with a median age of 4 months. Since ZAP-70 deficiency is an autosomal recessive form of CID with low to absent CD8^+^ T cells, most of the patients were initially diagnosed as SCID (16, 17). Interestingly, the study by Speckmann et al. found that having a genetic diagnosis was of limited value in predicting disease evolution (44).

Our results are mostly representative of the typical immune dysregulation in ZAP-70 deficiency, described as low to absent CD8^+^ T cell (97.9%), normal CD4+ T cell, normal B cell, and normal NK cell counts. However, immunoglobulin levels varied individually with the majority being within the normal range in most cases. A notable decline in PHA response (95%), specific antibody production (57%; with poor antipolysaccarid response), and TRECs (50%) were detected in patients with ZAP-70 deficiency, which was in line with previous studies (2, 45). The reduction in PHA response may reflect the presence of a CD4^+^ T cell dysfunction also, despite having normal frequency (46, 47). Therefore, it would be reasonable to screen for ZAP-70 deficiency in newborns with low TREC or low CD8^+^ T cell counts, consanguineous parents, and positive family history of CID. It is also crucial to highlight the fact, that TREC screening does not sufficiently identify these patients in newborn screening (48). The key lab finding, which presents early in ZAP-70 deficiency, is a low CD8 count. While TREC may be significantly reduced in individual patients, this probably worsens over time as exhibited by P25 (31). This is in line with the prospective evaluation that ZAP-70 has not been frequently picked up during the >10 year screening experience in the United States (49), despite a significant Mennonite population and the large number of reported ZAP-70 patients. Moreover, to affirm the conclusion for the impact of newborn screening, only TREC levels from the original newborn screening card can be taken into consideration.

Among the 49 patients with ZAP-70 deficiency, 32 unique *ZAP70* mutations were identified. Since the number of reported patients with ZAP-70 deficiency is small and the clinical pictures of the patients showed a striking heterogeneity, even in patients exhibiting a similar reduction of ZAP-70 expression, it was difficult to identify an association between the location and type of mutation with disease course or outcome (14). We did not find any correlation between clinical and laboratory findings and the reported mutation. Previous reports showed that the clinical consequences of ZAP-70 deficiency in patients with residual expression of this gene were attenuated, presenting as “Leaky ZAP-70 deficiency” as compared to patients presenting with a complete absence of ZAP-70 activity. Unlike patients with classical ZAP-70 deficiency, patients exhibiting residual expression of ZAP-70 have only mildly low numbers of CD8+ T cells with a small number of functional T cells (8), showing late-onset disease without signs of failure to thrive, severe infections (38) or autoimmunity (27). However, we did not find any significant differences between two the groups in clinical and laboratory findings. Likely, this result was due to the small sample size of the leaky group.

Allogenic HSCT seems to be the only curative therapy available for patients with ZAP-70 deficiency. Among the 25 patients who received HSCT, 22 patients (91.7%) ended up alive and well in a median follow up of 36 months; while, the survival rate for patients who did not undergo HSCT was 59.1% in median follow-up of 18 months. According to the Kaplan-Meier survival curve, HSCT significantly changes the patients' prognosis by decreasing the mortality rate. Unfortunately, it is not apparent whether the patients that had undergone HSCT were clinically less severe or the patients with severe disease had died before undergoing HSCT. Furthermore, some of the patients with “early deaths” may have been “transplant candidates” but judged to be “too sick” by their physician to undergo HSCT. We found that more patients who did not undergo HSCT and had died (*n* = 9) presented with malignancy, cardiovascular and neurologic involvement in comparison to alive non-HSCT patients (2 vs. 0 cases, 2 vs. 0 cases, and 3 vs. 1 cases, respectively). There were no immunological differences between the above groups. Nonetheless, further experimental studies are recommended for the development of new therapeutic strategies such as gene therapy for patients with ZAP-70 deficiency.

As described in the Results section, 8 out of the 12 patients (66%) with clinical complications post-HSCT were older than 6 months at the time of transplantation. It appears that younger patients undergoing HSCT, experience better outcomes and fewer complications; therefore, early screening and HSCT could lower the burden of the disease (48). However, no study has yet directly investigated the correlation between age at the time of transplantation and its outcome in ZAP-70-related CID. Therefore, more studies are required to support this hypothesis.

## Data Availability Statement

Publicly available datasets were analyzed in this study. This data can be found here: https://www.ncbi.nlm.nih.gov/pubmed/.

## Author Contributions

NS, MJ, MZ-D, MS, HM, FJ-N, SS, RY, HA, AA, and GA: substantial contributions to conception and design, acquisition of data, or analysis and interpretation of data. BL, NS, MJ, MZ-D, MS, HM, FJ-N, SS, RY, HA, AA, and GA: drafted the article or reviewed it critically for important intellectual content and given final approval of the version.

## Conflict of Interest

The authors declare that the research was conducted in the absence of any commercial or financial relationships that could be construed as a potential conflict of interest.
