# Supplementary figures and images for: Clinical, Immunological, and Genetic Features in 49 Patients With ZAP-70 Deficiency: A Systematic Review
Source: Front Immunol. 2020 May 5;11:831. doi: 10.3389/fimmu.2020.00831 (PMC7214800; doi:10.3389/fimmu.2020.00831)

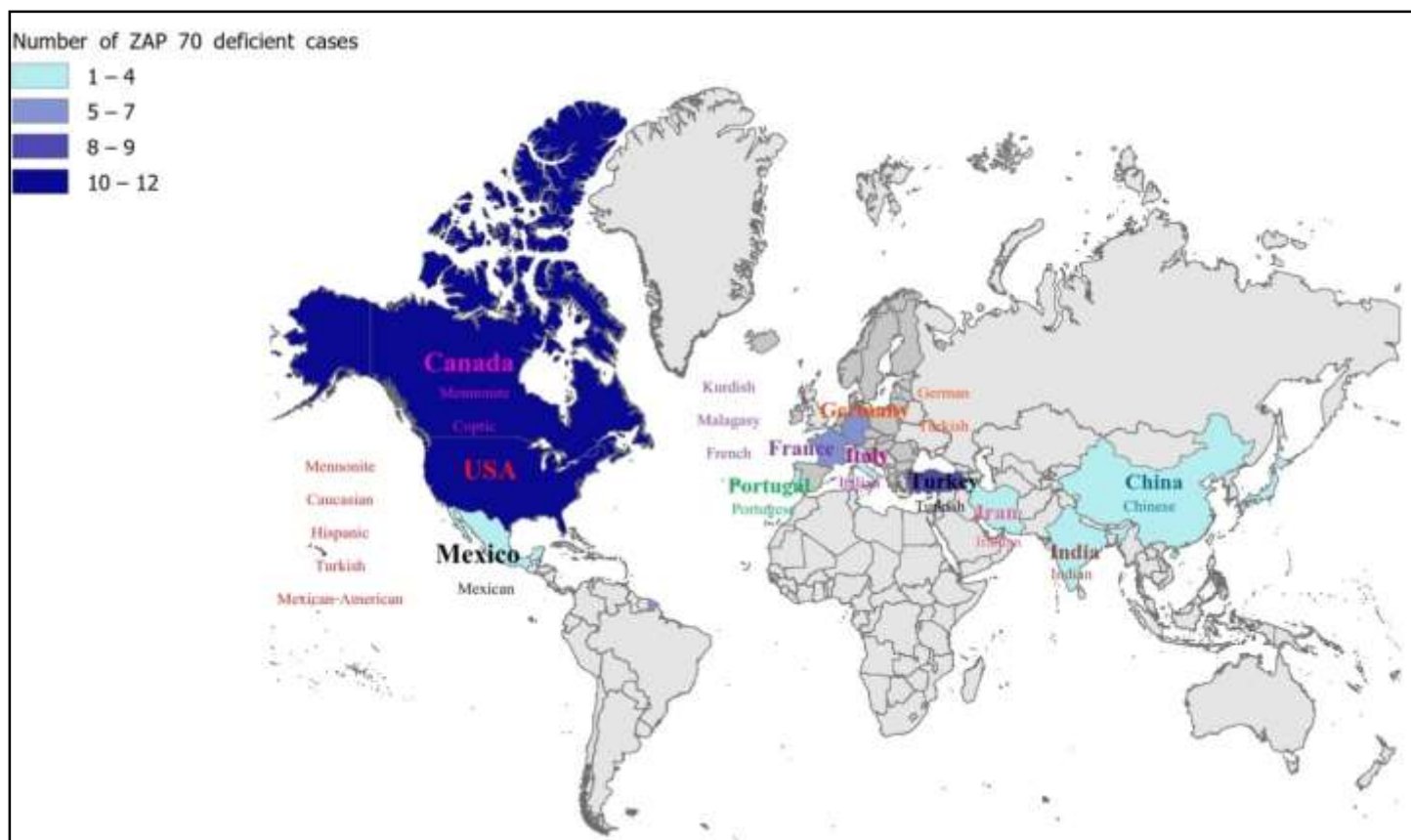

Figure S1: Geographical distribution of 49 reported patients with ZAP 70 deficiency.

Supplement: Supplementary file 1 [file Image_1.pdf]
