# Supplementary material for: Clinical, Immunological, and Genetic Features in 49 Patients With ZAP-70 Deficiency: A Systematic Review
Source: Front Immunol. 2020 May 5;11:831. doi: 10.3389/fimmu.2020.00831 (PMC7214800; doi:10.3389/fimmu.2020.00831)
Supplement: Supplementary file 2 [file Image_2.pdf]

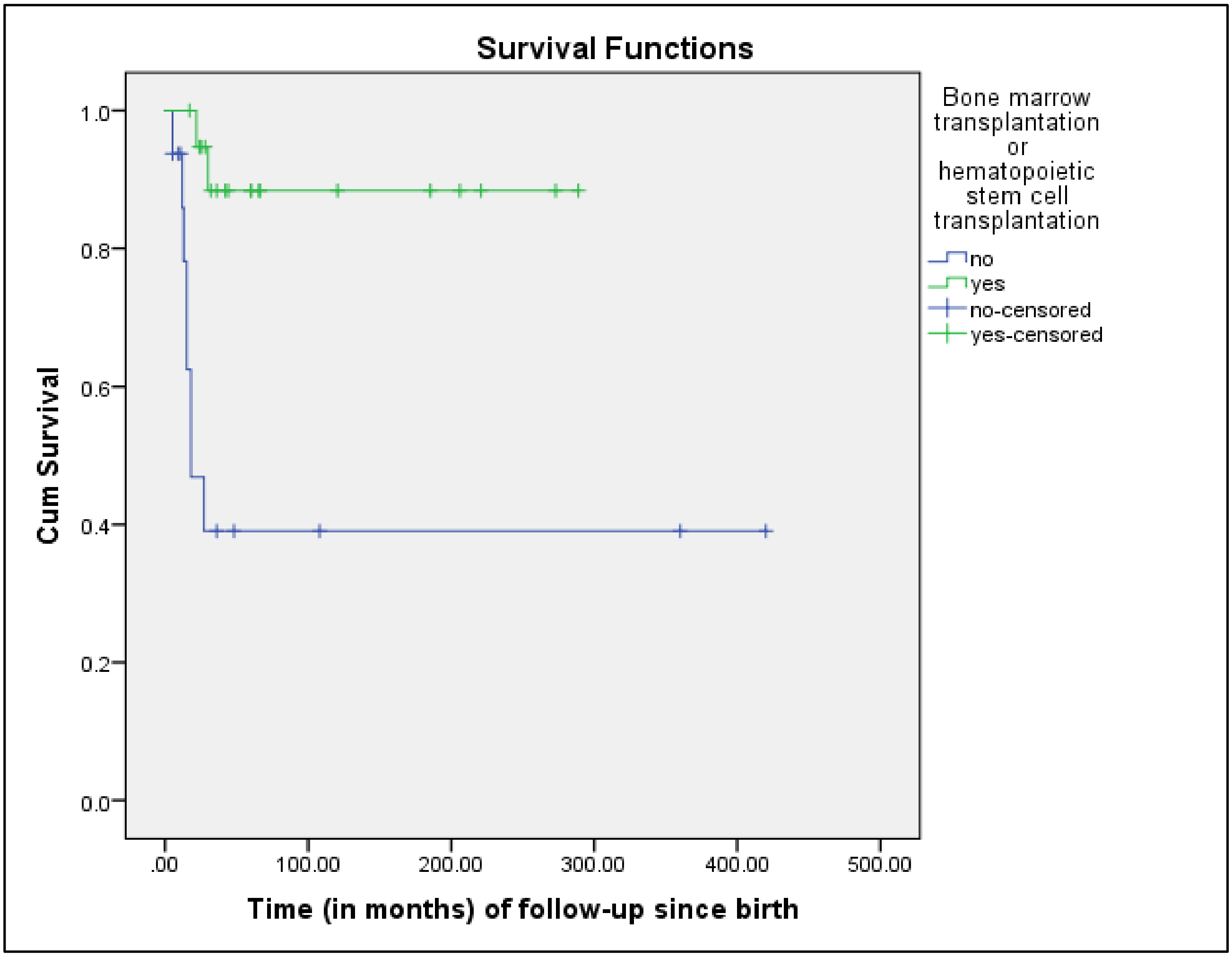

Figure S2: Kaplan-Meier plot for patients ZAP70 deficiency. Ninety percent of patients who underwent transplantation survived. According to the statistics, death was significantly lower in the HSCT group (*p*<0.001).
