# Supplementary material for: Clinical, Immunological, and Genetic Features in 49 Patients With ZAP-70 Deficiency: A Systematic Review
Source: Front Immunol. 2020 May 5;11:831. doi: 10.3389/fimmu.2020.00831 (PMC7214800; doi:10.3389/fimmu.2020.00831)
Supplement: Supplementary file 3 [file Table_1.pdf]

Table S1. The reported *ZAP70* gene mutations in patients with ZAP-70 deficiency

cDNA Mutation	Amino acid changes	Zygosity	Frequency (Family)	Ethnicity	Total number of patients	Mutation type	Reference
**c.508C>T**	p.R170C	Hom	1	Portugese	2 (P18-P19)	Missense	(4)
**c.837+121G>A**		Hom	1	Malagasy	1 (P17)	Splice site	(27)
**c.836_837delAT**		Hom	1		1 (P20)	InDel-Frameshift	(13)
**c.847C>T**	p.R283*	Hom	1	Indian	1 (P49)	Nonsense	(9)
**c.1010T>G**	p.L337A	Hom	1	Turkish	1 (P14)	Missense	(14)
**c.1153C>T**	p.R385C	Hom	1	Turkish	2 (P21-P22)	Missense	(28)
**c.1193C>T**	p.I398S	Hom	1	Turkish	2 (P28-P29)	Missense	(33)
**c.1272C>T**	p.G355G	Hom	1	Coptic	1 (P42)	Missense	(37)
**c.1483-1495del13**	p.A495fs*75	Hom	1	Kurdish	1 (P26)	InDel-Frameshift	(32)
**c.1504_1505insGC**	p.P502Afs*43	Hom	1	Turkish	1 (P30)	InDel-Frameshift	(33)
**c.1520C>T**	p.A507V	Hom	3	Turkish	3 (P15-P27-P31)	Missense	(14, 32, 34)
**c.1561G>A**	p.D521N	Hom	1	Irainian	1 (P43)	Missense	(39)
**c.1602C>T**	p.R465C	Hom	1	Caucasian	1 (P11)	Missense	(24)
**c.1603G>A**	p.R465H	Hom	1	Japanese	1 (P12)	Missense	(25)
**c.1624-11G>A**	p.K541_K542insLEQ	Hom	10	Mennonite	12 (P1-P6-P23-P25-P32 to 39)	Splice site	(6, 20, 22, 30, 31)
**c.1690T>C**	p.C564R	Hom	1	Turkish	1 (P16)	Missense	(14)
**c.1719_1731delTGACTGCTGGATC**	p.D574Tfs*10	Hom	2	Hispanic-German	2 (P5-P10)	InDel-Frameshift	(3, 23)
**c.1729C>T**	p.A507V	Hom	1	French	2 (P8-P9)	Missense	(21)
**c.1747C>T**	p.R514C	Hom	1	Turkish	1 (P24)	Missense	(29)
**c.183T>A**	p.I61N	Hom	1	Indian	1 (P44)	Missense	(35)
**c.1763C>A/ c.1624-11G>A**	p.S518R/p.K541_K542insLEQ	Comp Het	2	Mennonite	3 (P2-P3-P4)	Missense/Splice site	(17)
**c.37G>C/ c.1198C>T**	p.G13R / p.R400W	Comp Het	1	Mexican	1 (P45)	Missense/Missense	(7)
**c.448C>A/ c.1923A>T**	p.P80Q/ p.M572L	Comp Het	1	Japanese	1 (P7)	Missense/Missense	(12)
**c.574C>T / c.1079G>C**	p.R192W / p.R360P	Comp Het	1	Caucasian	2 (P40-P41)	Missense/Missense	(5)
**c.598-599delCT / c.847C>T**	p.L200fs*28/ p.R283*	Comp Het	1	Chinese	1 (P46)	InDel-Frameshift/Nonsense	(38)
**c.703-1G>A / c.1674G>A**	splice / p.M558I	Comp Het	1	Japanese	1 (P48)	Splice site/Missense	(8)

**c.733G>A / c.1505C>T**	p.G245R / p.P502L	Comp Het	1	Mexican-American	1 (P47)	Missense/Missense	(36)

Hom : homozygous, Comp Het: compound heterozygous, InDel: Insertion/deletion
